# Comparisons of lymphocytes profiles and inflammatory cytokines levels in blood of patients with differed severity of infection by human adenovirus type 7

**DOI:** 10.1186/s12879-023-08132-z

**Published:** 2023-03-22

**Authors:** Junping Sun, Xidong Ma, Mingyue Zhang, Mei Xie, Xingang Zhang, Xinjie Han, Xinfu Li, Enlu Zhou, Junyu Wang, Jianxin Wang

**Affiliations:** 1 Department of respiratory and critical care medicine, The Chinese PLA General Hospital, Heishanhu Road, Haidian Distrit, 100193 Beijing, China; 2 Department of respiratory and critical care medicine, West Beijing Medical District of People’s Liberation Army General Hospital, West Third Ring North Road, Haidian District, 100048 Beijing, China

**Keywords:** Human adenovirus type 7, Pneumonia, Lymphocyte subsets, Inflammatory cytokine, hypercytokinemia, CXCL10

## Abstract

**Background:**

Human adenovirus (HAdV) infection outbreak causes community-acquired pneumonia. Cellular immune dysfunction and hypercytokinemia play important roles in the pathogenesis of adenovirus respiratory infection. Some soluble factors in peripheral blood can assist in judging the virus-induced disease severity. The expression levels of inflammatory cytokines differ among patients with different disease severity. However, whether and how HAdV-7 infection influences the composition of blood immune cells and serum cytokine levels in patients at different disease stages, as well as the diagnosis values of these parameters, have rarely been intensively studied. We aimed to investigate lymphocytes profiles and cytokines levels in blood of patients at different disease stages upon human adenovirus type 7 (HAdV-7) infections, and explored the diagnosis values of the investigated parameters.

**Methods:**

Patients from two outbreaks of HAdV-7 in military of China were categorized into upper respiratory infection (URI) group, common pneumonia (CP) group and severe pneumonia (SP) group according to disease severity. Peripheral blood samples were subjected to routine laboratory tests, while flow cytometry and ELISA were used to measure the lymphocyte subsets and cytokines in blood, respectively. The receiver operating characteristic (ROC) curves were performed to examine the diagnostic of these blood parameters.

**Results:**

Signs of imbalanced lymphocytes composition and hypercytokinemia were observed in HAdV-7-infected patients. The percentages of CD3^+^ T cells and NK cells were significantly decreased along with the aggravation of the disease, particularly for NK cells and CD4^+^ T cells. The neutrophil to lymphocyte ratio (NLR) increased significantly in patients with more severe disease. In addition, the levels of serum CXCL10, IL-2 and TNF-α were positively correlated with disease severity, while reduced levels of IFN-γ and IL-10 were found in SP patients. Furthermore, analysis of ROC showed that multiple parameters including the percentage of blood CD3^+^ cells and serum CXCL10 level could predict the progression of HAdV-7 infection.

**Conclusion:**

Imbalance of immune state with hypercytokinemia occurred during HAdV-7 infection. The percentages of blood immune cells such as CD3^+^ T cells and the levels of serum cytokines such as CXCL10 showed potential diagnosis values in HAdV-7 infection.

**Supplementary Information:**

The online version contains supplementary material available at 10.1186/s12879-023-08132-z.

## Background

As double-stranded, nonenveloped DNA viruses belonging to the genus Mastadeno virus of the *Adenoviridae* family [1, 2], human Adenoviruses (HAdVs) are associated with a broad spectrum of clinical diseases, including acute respiratory and gastrointestinal infections in humans [3–5]. The main symptoms of adenovirus respiratory infection are cough, fever, and sore throat. HAdV infections occurred mainly in children and immunocompromised patients, owing to a lack of humoral immunity or impaired immunity [6, 7]. In addition, it occurs in densely populated crowds, especially new enlisted soldiers, which are prone to adenovirus infection [8].

Until recently, more than 100 different types of HAdV have been described and are designated with consecutive numbers following virus neutralization assay or genomic/bioinformatics analyses [4, 5]. Several genotypes of HAdV show tropism for the respiratory tract and are commonly associated with adenoviral respiratory infections [9, 10]. Among these genotypes, HAdV-3 and HAdV-7 are the most common causative agents worldwide [11, 12]. Particularly, HAdV-7 is the most pathogenic and can cause more severe respiratory illness and higher fatality rates than other HAdV types [13]. Outbreaks of HAdV-7 have been reported at military training bases, school clusters, and communities around the world [14–16]. For examples, HAdV-7 respiratory infections epidemic with 129 cases was reported in Wuhan Children’s Hospital, Hubei Province, China during the period between December 2018 and August 2019 [17] .

The host immune system developed multiple ways for recognition of adenovirus infection from the moment it enters the bloodstream [18]. In addition to altered generation and differentiation of immune cells upon HAdV infection, the release of cytokines/chemokines during an acute infection also occurs [19, 20]. Of note, some soluble factors in peripheral blood have been found to be able to predict the virus-induced disease progression accurately [21, 22]. It was reported that the expression levels of inflammatory cytokines differed among patients with different disease severity [1, 23]. However, whether and how HAdV-7 infection influences the composition of blood immune cells and serum cytokine levels in patients at different disease stages, as well as the diagnosis values of these parameters, have rarely been intensively studied.

In this study, we investigated the percentages of peripheral blood lymphocyte subset, neutrophil-to-lymphocyte ratio, and serum inflammatory cytokines levels in patients at different disease stages upon HAdV-7 respiratory infection, and evaluated the performances of these markers in predicting the disease progression. Our work provides valuable data to support appropriate clinical decision making and early identification of high-risk cases. .

## Methods

### Patients and study design

From January 2015 to March 2015, two outbreaks of HAdV-7 infection were reported in Ningxia province, China and Hubei province, China, where 219 patients and 218 patients were admitted to hospitals, respectively. Cases of HAdV-7-associated respiratory infection with laboratory-confirmed symptoms were initially enrolled in this study. Diagnoses were based on polymerase chain reaction findings from throat swab specimens. Sputum smear and culture of bacteria, fungi, mycoplasma, and chlamydia, as well as detection of Legionella antibody and related respiratory tract infection were performed to exclude patients with other pathogen infections. Patients with HIV infection, neutropenia, or diagnosis of pneumonia or other infectious diseases in the last 30 days, and those receiving immunosuppressive chemotherapy, were excluded.

Eventually, 79 cases in Ningxia province and 81 cases in Hubei province were enrolled in this study. According to guidelines for the diagnosis and treatment of adenovirus infection and chest radiographic findings, these cases were classified into two groups: upper respiratory infection (URI) and adenovirus pneumonia (AdP). The AdP group was further divided into common pneumonia (CP) group and severe pneumonia (SP) group according to the severity of pneumonia, as detailed below. This study complied with the necessary ethical guidelines and was approved by the Research Ethics Board of the People’s Liberation Army General Hospital (approval number S2022-419). All participants or their guardians signed an informed consent form before enrolment.

### Diagnostic criteria of adenovirus respiratory infection [24, 25]

Clinical and laboratory parameters were collected from all patients and used for diagnosis. All HAdV-7-infected cases had positive nucleic acid test results of throat swab specimens with the real-time quantitative PCR (RT-PCR) detection method. Diagnostic criteria for URI: No acute inflammatory infiltrating shadow was found in chest imaging and accompanied by any of the following manifestations: (1) acute onset of fever; (2) cough and expectoration, sore throat, fatigue, nausea, loss of appetite; (3) pharyngeal hyperemia, tonsil enlargement, surface visible patchy gray white secretion, bilateral cervical lymph nodes. Diagnostic criteria for CP: 1) New onset of cough or expectoration, or aggravation of existing symptoms of respiratory tract diseases, with or without purulent sputum, chest pain, dyspnea, or hemoptysis; 2)Fever; 3) Signs of pulmonary consolidation and/or moist rales; 4)Peripheral white blood cell count (WBC) > 10 × 10^9^/L or < 4 × 10^9^/L, with or without a left shift; 5) Chest radiograph showing new patchy infiltrates, lobar or segmental consolidation, ground glass opacities, or interstitial changes, with or without pleural effusion. Diagnosis can be established if a patient satisfies criterion 5 and any one condition of criterion1-4 and meanwhile, tuberculosis, pulmonary tumour, non-infectious interstitial lung disease, pulmonary edema, atelectasis, pulmonary embolism, pulmonary eosinophilia and pulmonary vasculitis are all excluded. The SP cases met the CP diagnostic criteria and had additionally any of the following conditions: (1) sustained high fever (> 39 ℃) more than 5 days, accompanied by frequent and severe irritating cough; (2) heart rate > 100 times/min and (or) respiratory rate > 30 times/min; (3) lung shadow progressed very quickly with multiple or single lobar/segment consolidation; (4) PaO_2_ < 70mmHg, and (or) SpO_2_ < 90%, while oxygen inhalation or mask oxygen can’t improve PaO_2_.

### Blood sample preparation

Blood samples were collected within 2 days of admission. For the analysis of lymphocyte subsets, 2 mL venous blood was stored in EDTA anticoagulant tube at room temperature for 0.5–2 h, and subjected to flow cytometry analysis after red blood cells lysis using ACK (Ammonium-Chloride-Potassium) lysing buffer. For the measurements of serum cytokines, 5 mL venous blood was placed in the coagulation tube at room temperature for 2 h, and supernatant was taken after centrifugation at 10 000 g/min for 15 min at 2–8 ℃. The supernatant samples were stored at − 80 ℃ until the assays. At the same time, blood routine tests were carried out on the second day of admission, and data on the absolute numbers of neutrophils and lymphocytes were collected. The ratio of neutrophils to lymphocytes (neutrophil to lymphocyte ratio; NLR) was calculated.

### Peripheral lymphocyte subsets analysis by flow cytometry

The percentages of peripheral blood lymphocyte subsets including CD3^+^ total T cells, CD3^+^CD4^+^ T cells, CD3^+^CD8^+^ T cells, NK cells (CD3^−^CD56^+^) and B cells (CD3^−^CD19^+^) were determined by flow cytometry. The following antibodies combinations were used: (1) for T-cell subsets, fluorescein-5-isothiocyanate-conjugated anti-CD45 (FITC-CD45) / phycoerythrin (RD1) - CD4/ECD- CD8/PC5- CD3; (2) for B cells, FITC- CD45/RD1- CD56 + PE-CD16/ECD-CD19/PC5-CD3; (3) for NK cells, FITC-CD3 / PE-CD16 + PE-CD56 / PC5-CD45. All antibodies were purchased from BD Biosciences (San Diego, CA, USA), and the flow staining followed the manufacturer’s instructions. Data were acquired with a flow cytometer (Model FC500, the Beckman Coulter company), and analyzed with the BD Biosciences supporting software (San Diego, CA, USA).

### Serum cytokine measurement by ELISA (enzyme-linked immunosorbent assay)

ELISA was used to measure The concentrations of serum cytokines including interferon-γ (IFN-γ), tumor necrosis factor –α (TNF-α), interleukin-10 (IL-10), interleukin-2 (IL-2), interleukin-17 A (IL-17 A) and IP-10 (IFN-γ inducible protein 10)/C–X–C motif chemokine 10 (CXCL10), were measured using the respective ELISA kits. The kits for measuring human IP-10/CXCL10, human IL-17 A, human IFN-γ, human IL-10, and human IL-2 were purchased from Wuhan Huamei, China. The TNF-α assay kit was from Siemens Healthcare Diagnostics Products Limited (Malvern, PA, USA). All the measurements and data analyses followed the manufacturers’ protocols.

### Statistical analysis

Continuous variables are expressed as mean ± standard deviation (SD) or median (interquartile range, IQR) as indicated. For continuous variables, paired *t*-test was used for normally distributed data, and nonparametric Mann Whitney U-test was used for non-normally distributed variables. For categorical variables, the differences between groups were tested by Chi-Square (χ2) test or Fisher exact test. For multiple comparisons between groups, analysis of variance or Kruskal Wallis test were used. Logistic regression coefficient method was used to test the correlation of each group. Receiver operating characteristic (ROC) curve was used to determine the evaluation and prediction value of relevant indicators. The sensitivity and specificity when the Yoden index was the largest were used to determine the best cutoff value. All analyses were performed with SPSS statistical software, version 22.0 (SPSS Inc; Chicago, IL, USA). A *P* value of less than 0.05 derived from a two-tailed test of all analyses was considered statistically significant.

## Results

### Characteristics of study subjects

All patients were military personnel on active duty in the Chinese army, and all the cases in this study were males. Among them, 79 cases were enrolled in Ningxia province, including 14 cases of SP, 41 cases of CP and 24 cases of URI. Their mean age was 19.72 ± 2.71 years. The other 81 cases were recruited in Hubei province, including 10 cases of SP, 30 cases of CP and 41 cases of URI. The mean age was 18.99 ± 1.45 years. Overall, a total of 95 AdP cases and 65 URI cases were enrolled, which accounted for 59.38% and 40.62% of all the cases, respectively. High-grade fever (39.09 ± 0.72 °C) was observed in most of the patients (98.13%). The highest temperature recorded was 41 °C, but temperatures did not differ significantly between groups. Most patients presented flu-like symptoms such as cough (80.63%) and sore throat (75.63%). Diarrhea (18.75%) was also observed and there was significant difference between groups (*P* < 0.01) (Table 1).

**Table 1:** The demographic and clinical data of patients in this study

Characteristic	All cases (n = 160)	URI (n = 65)	AdP (n = 95)
Age	19.36 ± 2.19	19.29 ± 2.26	19.39 ± 2.15
Fever	157(98.13)	63(96.92)	94(98.95)
Tmax(℃)	39.09 ± 0.72	39.10 ± 0.66	39.08 ± 0.76
Sore throat	121(75.63)	51(78.46)	70(73.68)
cough	129(80.63)	49(75.38)	80(84.21)
Diarrhea	30(18.75)	12(18.46)	18(18.95)**

Note: Except for the values of age and maximum body temperature, all other values are n (% of total); ** *P* < 0.01, between the groups of URI and AdP.

### HAdV-7-infection altered the frequencies of blood lymphocytes in patients

**Fig. 1:**
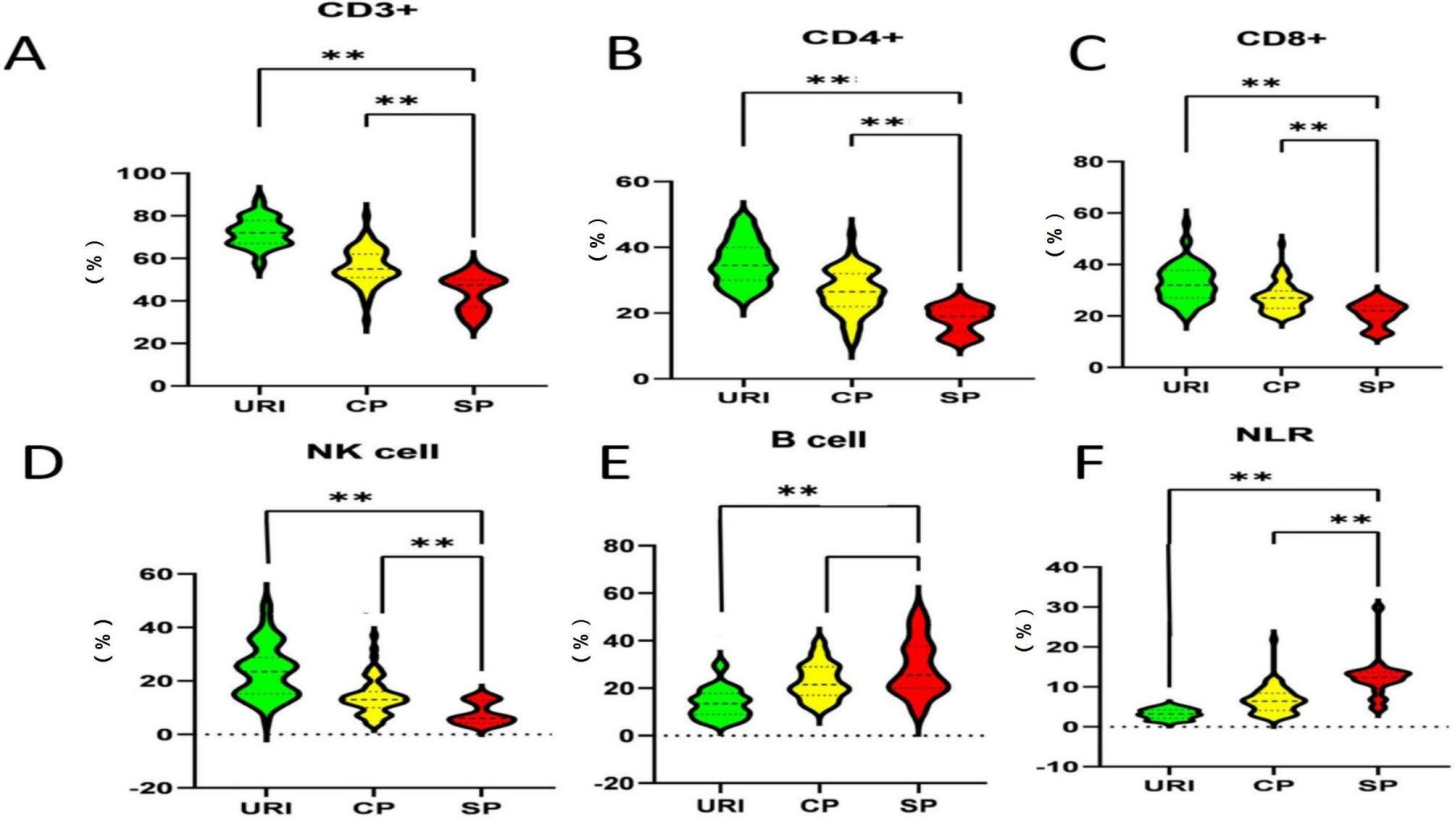
**The percentages of lymphocytes subsets at different disease stages upon HAdV-7 infection(A-F)** The percentages of CD3^+^ T cells (**A**), CD3^+^CD4^+^ T cells (**B**), CD3^+^CD8^+^ T cells (**C**), NK cells (**D**), and B cells (**E**) among the gated CD45^+^ lymphocytes in peripheral blood, as well as the NLR values (**F**), were compared among the patients of the indicated groups. URI, upper respiratory infection (n = 65); CP, common pneumonia (n = 71); SP, severe pneumonia (n = 24); NLR, neutrophils to lymphocytes ratio. *P < 0.05, ** *P* < 0.01, between the indicated groups

Flow cytometrical analysis of blood lymphocytes subsets were conducted to examine the impacts of HAdV-7 infection on the changes in distributions of major immune cells in peripheral blood (Fig. 1). Compared with the URI cases, the SP cases had significantly lowered percentage of CD3^+^ T cells among CD45^+^ leukocytes in blood (*P* < 0.001) (Fig. 1A). The percentage of CD4^+^ T cells among CD45^+^ cells (56.9%) in 91HAdV-7-infected patients was below the lower limit of the normal value. With the aggravation of the disease, the percentage of CD4^+^ T cells decreased more obviously, as the percentage of CD4^+^ T cells in SP cases was significantly lower than that of the CP cases (*P* < 0.001) (Fig. 1B). In addition, the Mean value of the percentage of CD8^+^ cells among CD45^+^ cells in SP cases were markedly lower than that in URI cases or CP cases (Fig. 1C). A correlation between disease progression and the decrease in percentage of NK cells (Fig. 1D) or the increase in percentage of B cells (Fig. 1E) were identified. Moreover, the NLR in HAdV-7-infected patients was also significantly higher than normal reference value and was associated with the severity of the illness (Fig. 1F). Furthermore, our logistical regression analysis suggested that the percentage of CD3^+^ cells and NLR values might be good indexes for diagnosis of HAdV-7 infection (Table S1). Collectively, these results indicated that HAdV-7 infection significantly altered the ratios of lymphocytes in peripheral blood.

### HAdV-7-infection induced hypercytokinemia in patients

**Fig. 2:**
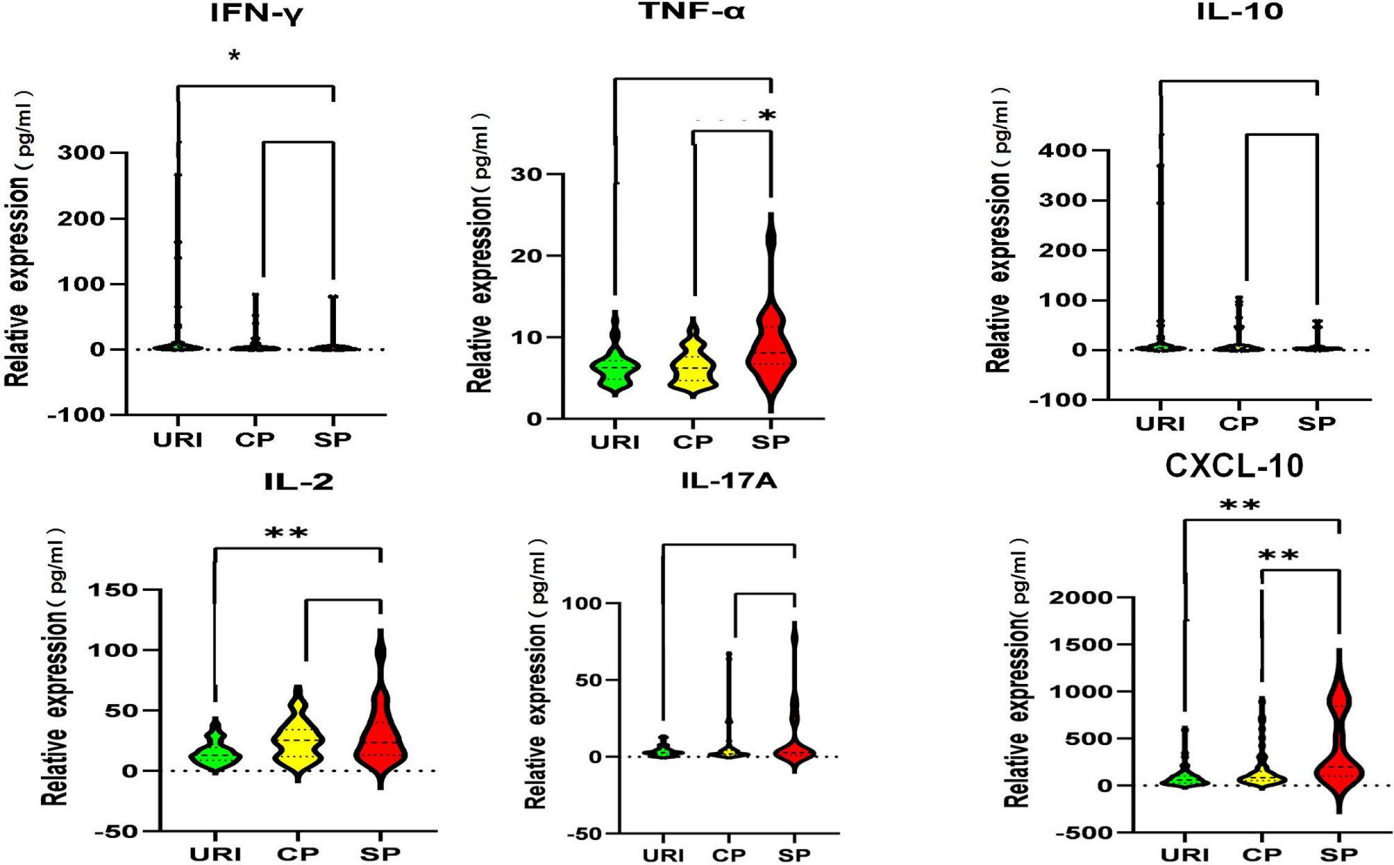
**The levels of serum cytokines from patients at different disease stages upon HAdV-7 infection** The levels of serum cytokines (pg/mL) including IFN-γ, TNF-α, IL-2, IL-17 A, IL-10 and CXCL10 from the patients of the indicated groups were measured by ELISA. *P < 0.05, ** *P* < 0.01, between the indicated groups

The cytokines levels in patient’s serum were associated with severity of illness. As shown in Fig. 2, the levels of TNF-α, IL-2, IL-17 A and CXCL10 were found to bear positive correlations with the severity of the disease. The level of TNF-α in SP cases was significantly higher than that in CP cases (*P* < 0.05). The level of IL-2 in AdP cases was significantly higher than that in URI cases (*P* < 0.001). Moreover, the levels of serum CXCL10 between URI cases and AdP cases also had significantly differences (*P* < 0.001), while the SP cases had a significantly higher level of CXCL10 than the CP cases (*P* < 0.001). However, the level of serum IL-10 and IFN-γ did not rise like other cytokines, but appeared to be reduced along with disease aggravation upon HAdV-7 infection. In addition, the levels of serum IL-2 and CXCL10 seemed to be markers that can be used for diagnosis of HAdV-7 infection according to the diagnosis logistic regression results (Table S2). Taken together, hypercytokinemia was observed in the patients infected with HAdV-7.

### Performance of the frequencies of lymphocyte subsets in predicting the disease progression of HAdV-7 infection

**Fig. 3:**
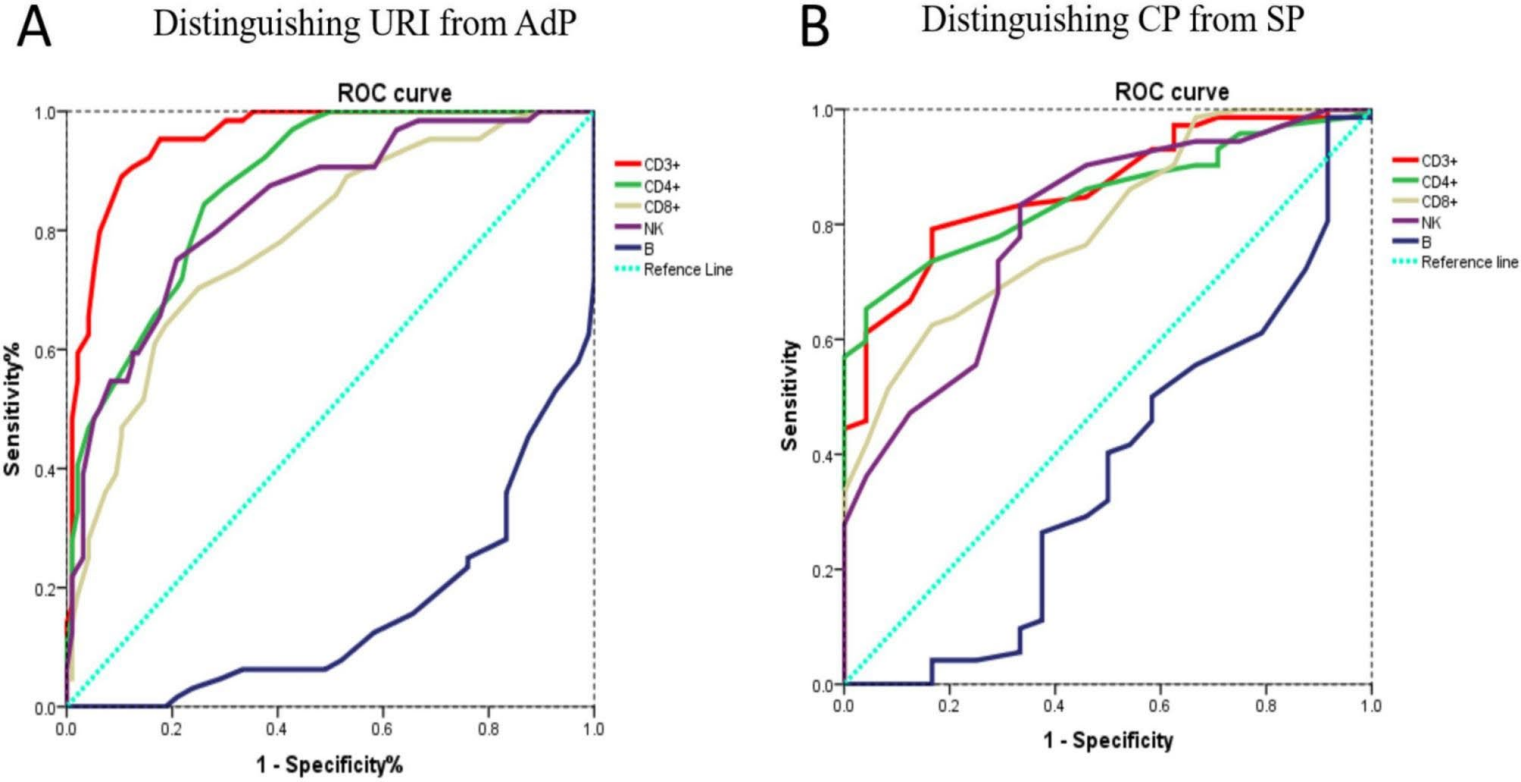
**The performance of the percentage of peripheral blood lymphocytes in predicting disease progression** ROC curves of the indicated different parameters (the percentage of blood CD3^+^ cells, CD4^+^ cells, CD8^+^ cells, NK cells, and B cells) in predicting the progression of disease from URI to AdP (**A**) and from CP to SP (**B**)

The performances of the frequencies of blood lymphocytes populations in predicting HAdV-7 infection induced disease progression from URI to AdP (Table S3 and Fig. 3A) and from CP to SP were evaluated (Table S4 and Fig. 3B). The percentage of CD3^+^ cells was found to be with a relatively higher accuracy in predicting the progression of HAdV-7 infection: 65.5% was the optimal threshold for prediction of URI to AdP, with a sensitivity of 89.1% and specificity of 89.6%. The area under curve in predicting URI to AdP is 0.954 (Table S3 and Fig. 3A). The percentage of CD3^+^ cells was found to be also good in predicting the progression from CP to SP: 50.5% was the optimal threshold, with a sensitivity of 79.2% and specificity of 83.3% (Table S4 and Fig. 3B).

### Performance of the serum cytokines levels in predicting the disease progression of HAdV-7 infection

**Fig. 4:**
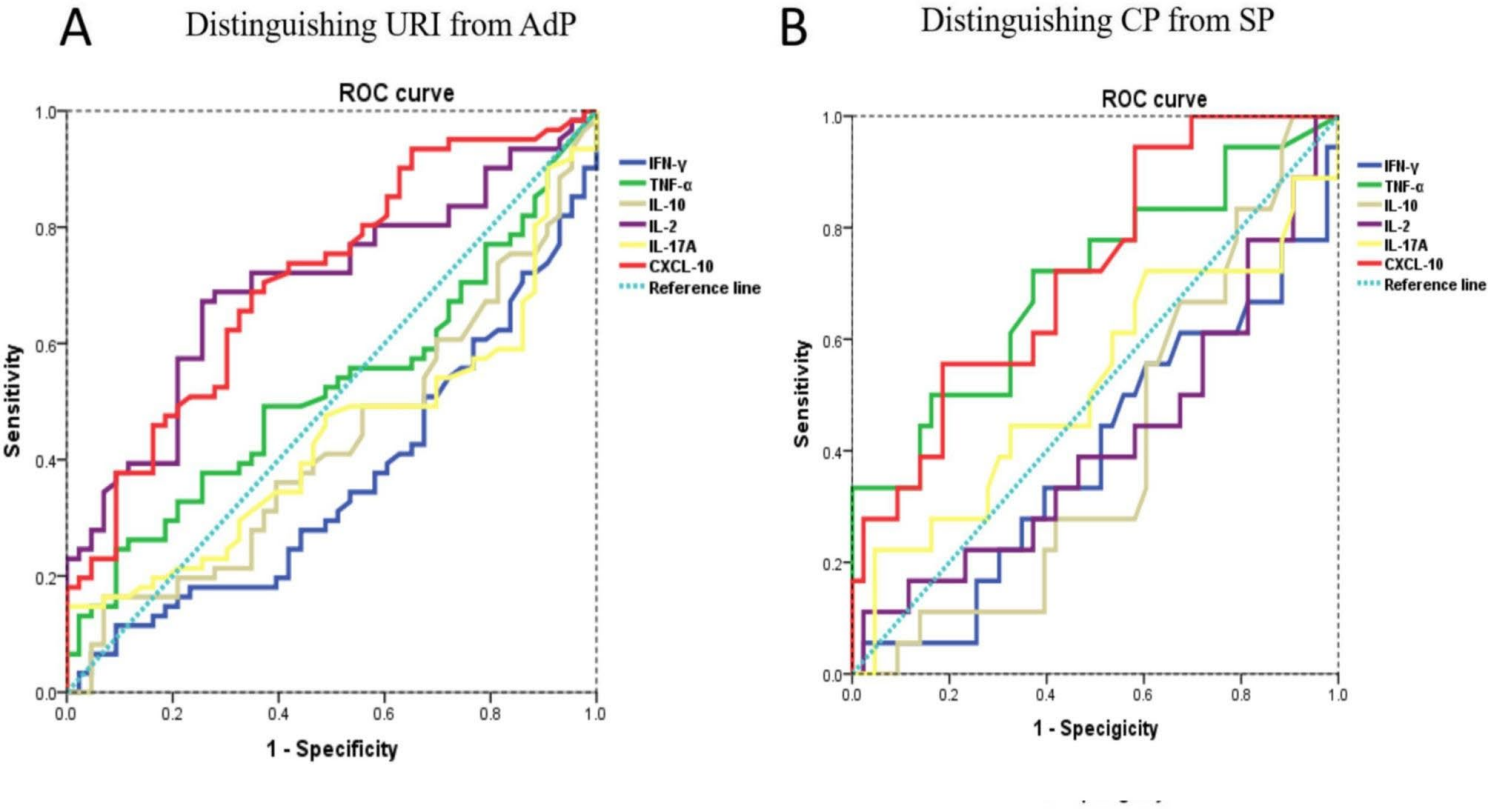
**The performance of the level of blood cytokine in predicting disease progression** ROC curves of the indicated different parameters (the level of serum CXCL10, IFN-γ, TNF-α, IL-2, IL-17 A, and IL-10) in predicting the progression of disease from URI to AdP (**A**) and from CP to SP (**B**)

The performances of the levels of blood cytokines in predicting HAdV-7 infection induced disease progression from URI to AdP (Table S5 and Fig. 4A) and from CP to SP (Table S6 and Fig. 4B) were also evaluated. As shown in Fig. 4A, CXCL10 and IL-2 showed relative better performance in predicting URI to AdP than other soluble factors. A CXCL10 concentration of 77.82 pg/mL was determined to be the best cutoff value, with an area under the curve (AUC) of 0.710, sensitivity of 68.9%, and specificity of 65.1%. For IL-2 concentration, the best cutoff value was 20.07 pg/mL, with an AUC of 0.699, sensitivity of 67.2%, and specificity of 74.4% (Table S5). The parameters CXCL10 and TNF-α were considered to be highly accurate for predicting the progression of CP to SP (Fig. 4B). The AUC for CXCL10 was 0.721 with 55.6% sensitivity and 81.4% specificity; while the AUC for TNF-α was 0.702 with 72.2% sensitivity and 62.8% specificity (Table S6).

## Discussion

Experiences from the ongoing global pandemic caused by severe acute respiratory coronavirus-2 (SARS-CoV-2) infection highlight the importance of distinct diagnosis, treatments, and prognosis of a particular infectious pathogen [26, 27]. Similarly, accurately differentiating HAdV infection in a timely manner and appropriate prognosis of patients with HAdV infection are crucial for both clinicians and epidemiologists. Here, we investigated the changes of both the frequencies of lymphocyte subsets and the cytokine levels in peripheral blood of patients during the disease progression upon HAdV-7 infection, and explored the diagnosis performance of these changed parameters in predicting the severity of HAdV-7 infection.

Significantly altered compositions of lymphocytes in peripheral blood were observed in patients with different disease severity of HAdV-7 infection, implying possible immune system damage due to imbalance of immune state in these patients with more aggravated disease. We found that the percentages of CD3^+^, CD4^+^ and NK cells were decreased and that of B lymphocyte was increased, which was more evident and significant in SP cases. CD4^+^ cells and CD8^+^ cells play an important role in the process of removing the adenovirus from the respiratory tract. CD4^+^ cells can secrete IFN-γ, TNF-α, IL-17 A and other different cytokines to participate in the antiviral immunity [28, 29]. CD8^+^ cells can direct lyse the infected cells and release proinflammatory cytokines such as IFN-γ and TNF-α [30]. The decreased frequencies of T cells in blood might be due to death of existed T cells during the combating against the HAdV-7 virus or suppression of T cell biogenesis by the virus. In addition, the enhancement of antibody-associated immunity, which caused more production of B cells, could also lead to a reduced percentage of T cells in blood. Different from our findings, Chen et al. reported that patients with severe HAdV-55 infections had significantly higher levels of IL-17^+^CD4^+^ cells and decreased levels of IL-17^+^CD8^+^ cells in blood [31]. These might be due to distinct pathogenesis of different adenovirus or the inherent disparities among different cohorts of patients. In addition, NK cell of the patients with HAdV-7 infection decreased significantly in this study, which was closely related to the severity of pneumonia. Although patients with different severities of disease due to HAdV-7 infection had significantly different immune responses, our results altogether suggest that monitoring the percentage of blood NK cells has clinical significance to assess the severity of pneumonia.

In our study, hypercytokinemia was observed in patients infected with HAdV-7. Hypercytokinemia (also known as a “cytokine storm”) is characterized by the over production of various proinflammatory cytokines, and plays an important role in the pathogenesis of virus infection [32]. We showed that the cytokines levels in patient were associated with deterioration of HAdV-7 infection. CXCL10 is a proinflammatory cytokine that is secreted by a variety of cells. It was elevated in patients with HAdV-7 infection, particularly in patients with SP. The high elevation of CXCL10 has been reported in the plasma of patients with SARS-CoV, avian influenza H5N1, and swine-origin influenza virus -infected macrophages [33–35]. Wenxin et al. found that during HAdV-7 infection, CXCL10 was secreted from both macrophages and epithelial cells [36], suggesting a comprehensive evaluation of CXCL10 in both peripheral blood and local infected tissues might be more helpful to predict the severity of HAdV-7 infection-caused diseases.

IL-17 A stimulates proinflammatory chemokine and recruits neutrophils into the airway, thus playing a significantly defensive role in various infections [37]. In our study, IL-17 A was elevated in HAdV-7 infected patients and seemed positively correlated with the severity of pneumonia. In line with our findings, in patients with H7N9 infection, high plasma concentrations of IL-17 A were also identified [38], suggesting that IL-17 A and the Th17 pathway are promising targets for developing drugs to attenuate virus infection-associated disease progression. Moreover, inconsistent with other reports [19, 31], IFN-γ in our study did not show a significantly higher level in blood of patients with more severe disease status, possibly reflecting the weaker antiviral activities in patients with AdSP due to the lack of IFN-γ at certain disease stages. Notably, we found that the levels of serum cytokines such as CXCL10, IL-2 and TNF-α can be biomarkers that can be used to predict disease progression in HAdV-7-infected patients to certain extent. Taken together, these data provide valuable insights on measuring blood parameters to identify the patients at risk of more severe disease.

The current investigation has several limitations. The major limitation is its sample size of patients. The patients included in this study were drawn from two cohorts and thus were subject to referral bias. These results need to be confirmed by studies of other outbreaks of HAdV-7. In addition, since our study was limited to infection by one HAdV-7 serotype, whether the results can be applicable to other serotypes needs to be further studied. Furthermore, peripheral blood was the only sample type, and data from infected organs like lungs and lymph nodes are supposed to be more valuable and informative. These results can help tell the changes in lymphocyte populations and cytokine levels were due to changes in absolute numbers or cell migration, and help distinguish the status of local inflammation to better predict disease progression. Taken together, the observation on the dynamic changes of the parameters and future multicenter validation of these parameters from a large sample size of patients would provide more supportive results.

## Conclusion

In conclusion, patients with HAdV-7 respiratory infection showed signs of imbalanced immune state, as evidenced by reduced frequencies of T cells and NK cells and increased frequencies of B cells in blood. The levels of serum cytokines including CXCL10, IL-2, and TNF-α appeared to be correlated with the severity of pneumonia. Monitoring lymphocyte subgroup and the levels of cytokines might help the judgments on the diagnosis and development of HAdV-7 infection-associated diseases.

## Electronic supplementary material

Below is the link to the electronic supplementary material.

Supplementary Material 1

## Data Availability

All data generated or analysed during this study are included in this published article and its supplementary information files.

## Abbreviations

HadV: Human adenovirus
HAdV-7: Human adenovirus type 7
URI: Upper respiratory infection
CP: Common pneumonia
SP: Severe pneumonia
ROC: Receiver operating characteristic
NLR: Neutrophil to lymphocyte ratio
AdP: Adenovirus pneumonia
RT-PCR: Real-time quantitative PCR
WBC: White blood cell count
IFN-γ: Interferon-γ
TNF-α: Tumor necrosis factor –α
IL-10: Interleukin-10
IL-2: Interleukin-2
IL-17A: Interleukin-17 A
CXCL10: C–X–C motif chemokine 10
SD: Standard deviation
IQR: Interquartile range
SARS-CoV-2: Rsevere acute respiratory coronavirus-2
