# Supplementary material for: Comparisons of lymphocytes profiles and inflammatory cytokines levels in blood of patients with differed severity of infection by human adenovirus type 7
Source: BMC Infect Dis. 2023 Mar 22;23:174. doi: 10.1186/s12879-023-08132-z (PMC10031703; doi:10.1186/s12879-023-08132-z)
Supplement: Supplementary file 1 — Supplementary Material 1 [file 12879_2023_8132_MOESM1_ESM.docx]

**Table S1. Correlation between the percentage of blood lymphocyte subsets and diagnosis Logistic regression results.**

| Index | B value | SE | *P* value | *OR* | 95%CI |
| --- | --- | --- | --- | --- | --- |
| CD3+ | -0.436 | 0.184 | 0.018 | 0.646 | 0.450-0.927 |
| CD4+ | -0.493 | 0.263 | 0.061 | 0.611 | 0.365-1.024 |
| CD8+ | -0.429 | 0.220 | 0.052 | 0.651 | 0.423-1.003 |
| NK | -0.457 | 0.240 | 0.057 | 0.633 | 0.395-1.015 |
| B | -0.092 | 0.140 | 0.514 | 0.912 | 0.693-1.202 |
| NLR | 2.275 | 1.079 | 0.033 | 9.732 | 1.173-80.711 |

**Table S2. Correlation between the serum cytokine levels and diagnosis Logistic regression results.**

| Cytokine | Β value | SE | *P* value | *OR* | *95%CI* |
| --- | --- | --- | --- | --- | --- |
| INF-γ | -0.021 | 0.018 | 0.233 | 0.979 | 0.945-1.014 |
| TNF-α | 0.074 | 0.121 | 0.538 | 1.077 | 0.850-1.365 |
| IL-10 | -0.004 | 0.007 | 0.625 | 0.996 | 0.982-1.011 |
| IL-17A | 0.020 | 0.022 | 0.587 | 1.073 | 1.028-1.121 |
| IL-2 | 0.071 | 0.037 | 0.001 | 1.020 | 0.949-1.098 |
| CXCL10 | 0.006 | 0.002 | 0.020 | 1.006 | 1.001-1.010 |

| Variables | ROC | *P* value | 95% CI | Cutoff value  (pg/mL) | Susceptibility (%) | Specificity  (%) |
| --- | --- | --- | --- | --- | --- | --- |
| CD3+ | 0.954 | 0.000 | 0.924-0.984 | 65.5 | 89.1 | 89.6 |
| CD4+ | 0.875 | 0.000 | 0.824-0.926 | 28.5 | 84.4 | 74 |
| CD8+ | 0.785 | 0.000 | 0.714-0.857 | 29.5 | 64.1% | 81.2 |
| NK | 0.839 | 0.000 | 0.777-0.901 | 15.5 | 75 | 79.2 |
| B | 0.159 | 0.000 | 0.097-0.221 | 10.5 | 62.5 | 1.0 |

**Table S3. Performance of the percentage of blood lymphocyte subset in predicting the progression of URI to AdP.**

**Table S4. Performance of the percentage of lymphocyte subset in predicting the progression of CP to SP.**

| Variables | AUC | *P* value | 95%CI | Cutoff value (pg/mL) | Susceptibility (%) | Specificity (%) |
| --- | --- | --- | --- | --- | --- | --- |
| CD3+ | 0.862 | 0.000 | 0.786-0.937 | 50.5 | 79.2 | 83.3 |
| CD4+ | 0.847 | 0.000 | 0.772-0.922 | 23.5 | 65.3 | 96.8 |
| CD8+ | 0.799 | 0.000 | 0.706-0.893 | 25.5 | 62.5 | 83.3 |
| NK | 0.795 | 0.000 | 0.693-0.896 | 7.5 | 83.3 | 66.7 |
| B | 0.370 | 0.056 | 0.232-0.507 | 11.5 | 98.6 | 8.3 |

**Table S5. Performance of the serum cytokine levels in predicting the progression of URI to AdP.**

| Variables | AUC | *P* value | 95%CI | Cutoff value  (pg/mL) | Susceptibility (%) | Specificity (%) |
| --- | --- | --- | --- | --- | --- | --- |
| INF-γ | 0.362 | 0.017 | 0.256-0.469 | 11.685 | 11.5 | 90.7 |
| TNF-α | 0.522 | .709 | 0.410-0.633 | 8.675 | 24.6 | 90.7 |
| IL-10 | 0.429 | 0.222 | 0.319-0.540 | 35.26 | 16.4 | 93 |
| IL-17A | 0.435 | 0.262 | 0.324-0.546 | 17.82 | 14.8 | 100 |
| IL-2 | 0.699 | 0.001 | 0.599-0.800 | 20.07 | 67.2 | 74.4 |
| CXCL10 | 0.710 | 0.000 | 0.611-0.810 | 77.82 | 68.9 | 65.1 |

**Table S6. Performance of the serum cytokine levels in predicting the progression of CP to SP.**

| Variables | ROC | *P* value | 95%CI | Cutoff value  (pg/mL) | Susceptibility (%) | Specificity (%) |
| --- | --- | --- | --- | --- | --- | --- |
| INF-γ | 0.394 | 0.195 | 0.235-0.553 | 66.505 | 5.6 | 97.7 |
| TNF-α | 0.702 | 0.013 | 0.550-0.855 | 6.755 | 72.2 | 62.8 |
| IL-10 | 0.403 | 0.236 | 0.256-0.551 | 0.59 | 100 | 9.3 |
| IL-17A | 0.520 | 0.806 | 0.347-0.693 | 24.475 | 22.2 | 95.3 |
| IL-2 | 0.416 | 0.304 | 0.251-0.581 | 59.03 | 11.11 | 97.7 |
| CXCL10 | 0.721 | 0.007 | 0.585-0.857 | 182.055 | 55.6 | 81.4 |
